# Supplementary figures and images for: Coming Clean and Avoiding Bubble Trouble–Using Detergents Wisely in the Purification of Membrane Proteins for Cryo-EM Studies
Source: Biomolecules. 2025 Sep 12;15(9):1315. doi: 10.3390/biom15091315 (PMC12466997; doi:10.3390/biom15091315)

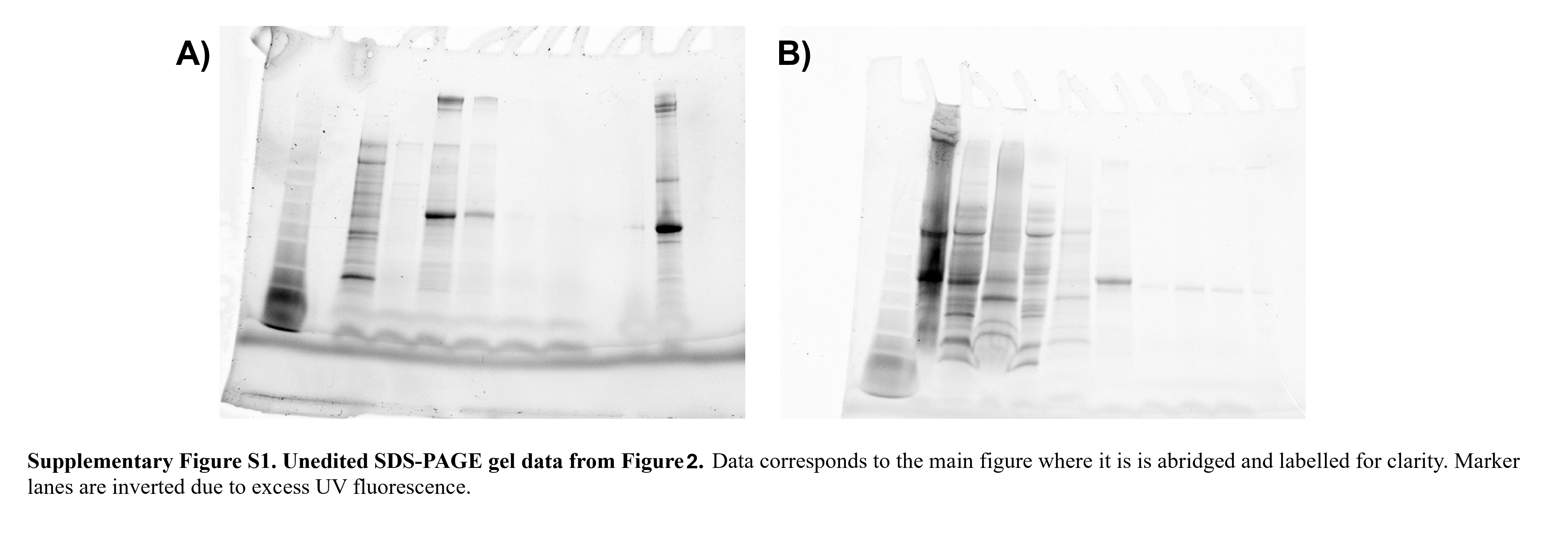

Supplement: Supplementary file 1 [file biomolecules-15-01315-s001.zip › FigureS1.tif]

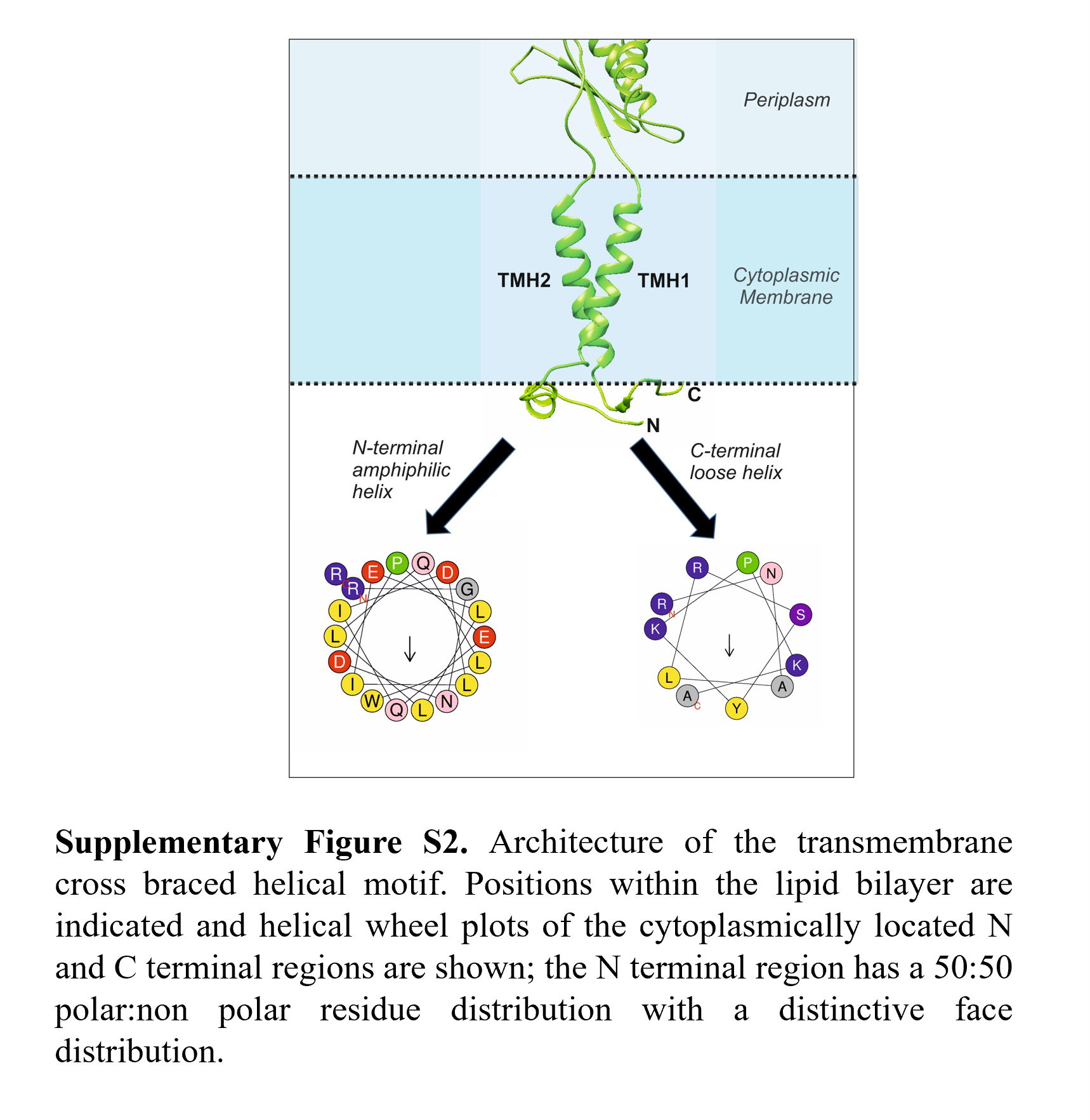

Supplement: Supplementary file 1 [file biomolecules-15-01315-s001.zip › FigureS2.tif]
